# Supplementary material for: Isotopic Tracers Unveil Distinct Fates for Nitrogen Sources during Wine Fermentation with Two Non-Saccharomyces Strains
Source: Microorganisms. 2020 Jun 16;8(6):904. doi: 10.3390/microorganisms8060904 (PMC7356982; doi:10.3390/microorganisms8060904)
Supplement: Supplementary file 1 [file microorganisms-08-00904-s001.zip › Supplementary_figures.pptx]

## Slide 1
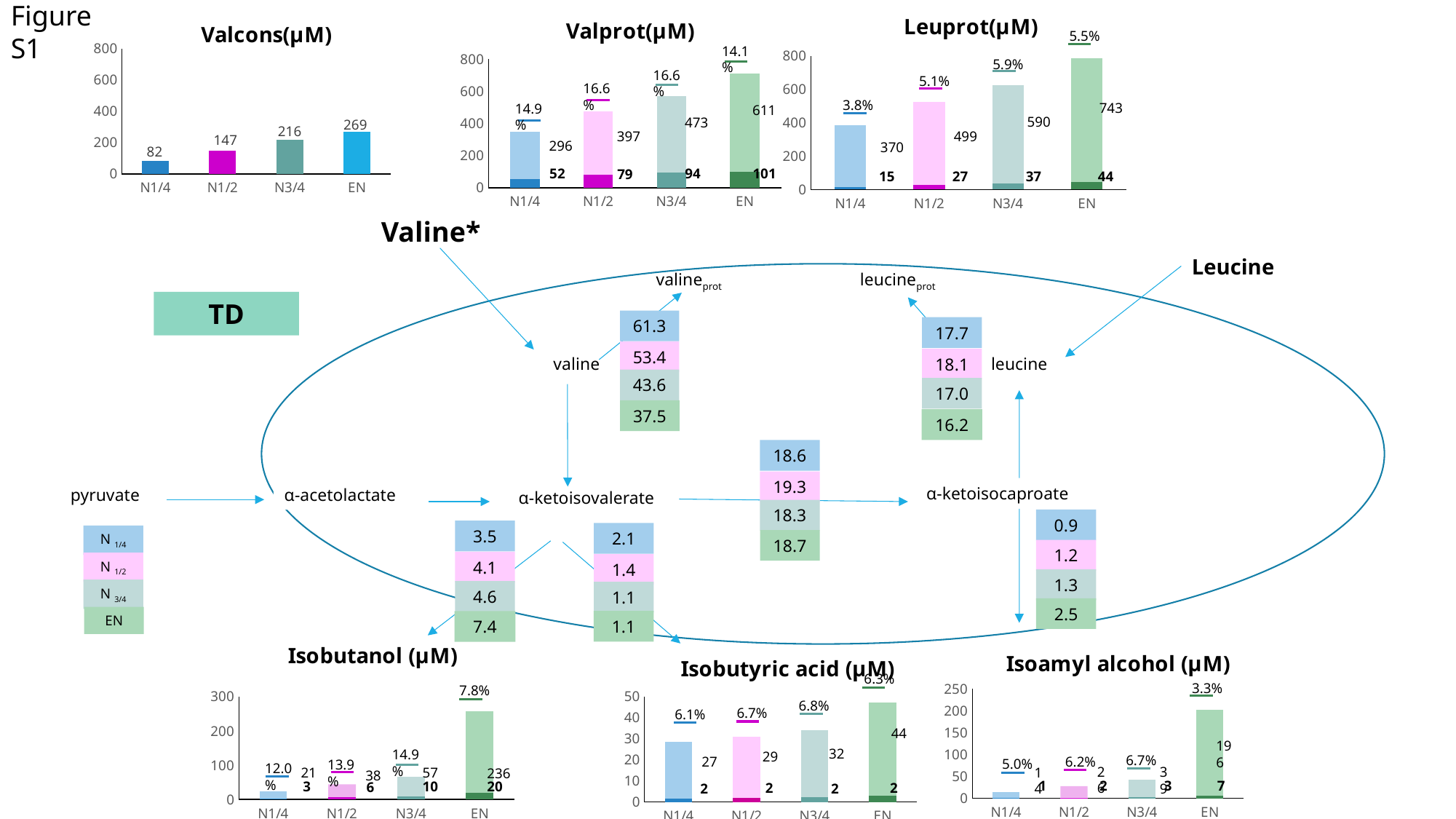

### Chart: Leuprot(µM)
| Category | Labeled | Unlabeled |
|---|---|---|
| N1/4 | 14.528628352197765 | 370.33569885833913 |
| N1/2 | 26.732493663561275 | 498.9802627486564 |
| N3/4 | 36.84127860897975 | 590.2443147353563 |
| EN | 43.63304873308469 | 743.2569194018962 |5.5%
5.9%
5.1%
3.8%
743
590
499
370
15
37
27
44
### Chart: Valprot(µM)
| Category | Labeled | Unlabeled |
|---|---|---|
| N1/4 | 51.75558346805186 | 296.29810495193374 |
| N1/2 | 78.69292196702648 | 396.6494034151933 |
| N3/4 | 94.27424889992959 | 473.1301982859241 |
| EN | 100.65018838137645 | 610.9099981253498 |14.1%
16.6%
16.6%
14.9%
611
473
397
296
101
52
94
79
Figure S1
### Chart: Valcons(µM)
| Category | cons uM |
|---|---|
| N1/4 | 81.98326339897625 |
| N1/2 | 147.48507654490612 |
| N3/4 | 216.23352101495212 |
| EN | 268.51282051282055 |Valine*
Leucine
valineprot
leucineprot
TD
61.3
53.4
43.6
37.5
17.7
18.1
17.0
16.2
valine
leucine
18.6
19.3
18.3
18.7
α-ketoisocaproate
pyruvate
α-acetolactate
α-ketoisovalerate
0.9
1.2
1.3
2.5
3.5
4.1
4.6
7.4
2.1
1.4
1.1
1.1
N 1/4
N 1/2
N 3/4
EN
### Chart: Isoamyl alcohol (µM)
| Category | labelled | unlabelled |
|---|---|---|
| N1/4 | 0.7245205157297855 | 13.780394714095749 |
| N1/2 | 1.7477559102902895 | 26.26115290846435 |
| N3/4 | 2.825872667130226 | 39.13214763175955 |
| EN | 6.609844730989846 | 195.83562941877577 |3.3%
196
6.7%
6.2%
5.0%
26
39
14
2
7
1
3
### Chart: Isobutanol (µM)
| Category | labelled | unlabelled |
|---|---|---|
| N1/4 | 2.8714339817810943 | 21.027309104203876 |
| N1/2 | 6.100164312744361 | 37.864983887215004 |
| N3/4 | 9.99490952539587 | 57.08502017524755 |
| EN | 19.96022579550792 | 236.10424881011008 |
### Chart: Isobutyric acid (µM)
| Category | labelled | unlabelled |
|---|---|---|
| N1/4 | 1.749596076201878 | 26.815237820971642 |
| N1/2 | 2.0842875115472412 | 28.93189569600099 |
| N3/4 | 2.315779882113411 | 31.59022132013272 |
| EN | 2.9793485166192495 | 44.19956702762857 |6.3%
7.8%
6.8%
6.7%
6.1%
44
32
14.9%
29
27
13.9%
12.0%
21
57
236
38
3
10
20
6
2
2
2
2

## Slide 2
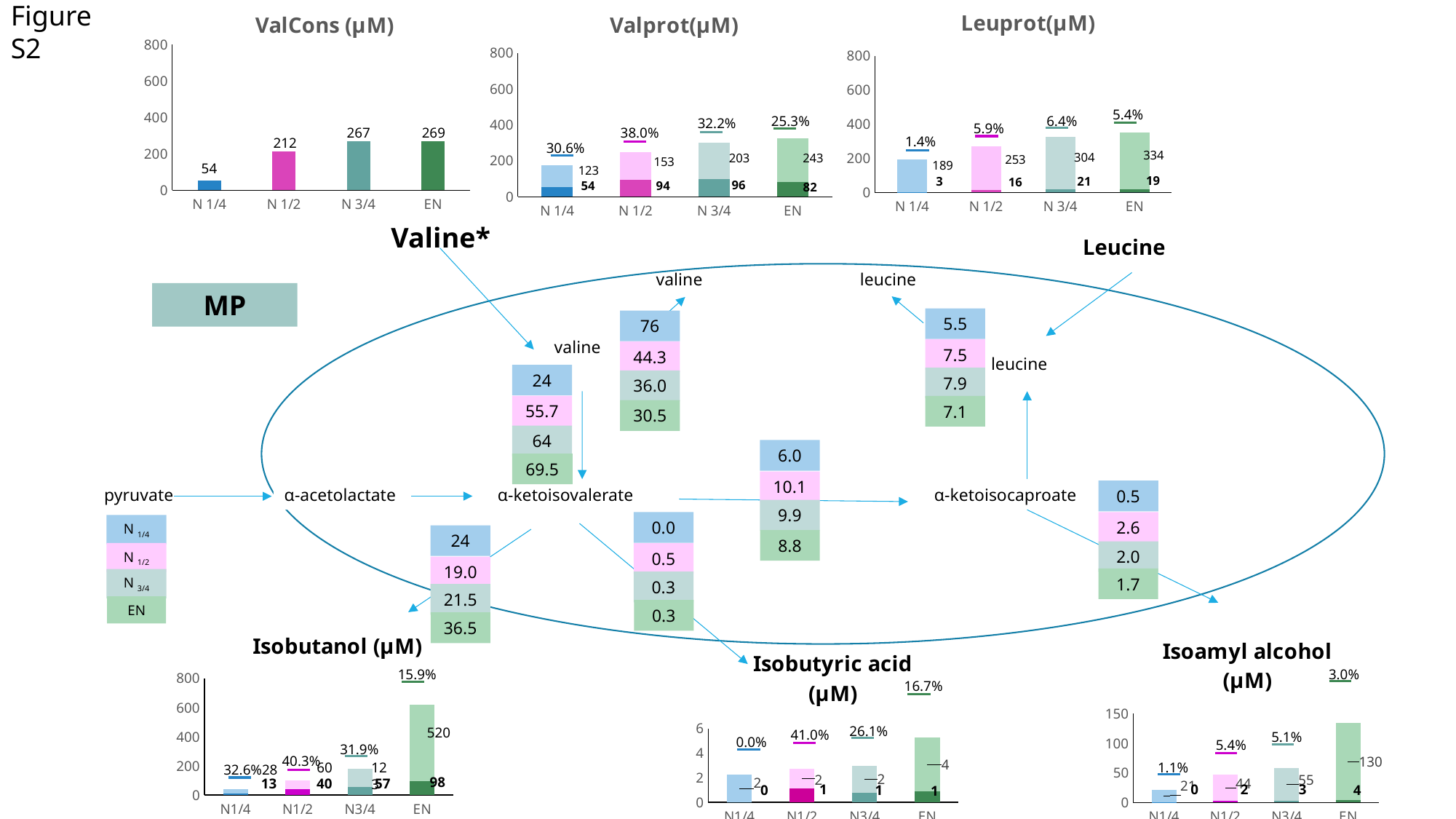

### Chart: ValCons (µM)
| Category | consumed |
|---|---|
| N 1/4 | 54.45713275897787 |
| N 1/2 | 211.86783910326713 |
| N 3/4 | 267.35663503456914 |
| EN | 268.51282051282055 |
### Chart: Valprot(µM)
| Category | labelled val | proteinogenic |
|---|---|---|
| N 1/4 | 54.173574050633185 | 122.77750891730142 |
| N 1/2 | 94.1023597445786 | 153.2425206459552 |
| N 3/4 | 96.43560048472527 | 203.0073446626905 |
| EN | 82.3660332346474 | 243.12708367761266 |
### Chart: Leuprot(µM)
| Category | 作图用 | labelled Leu | proteinogenic Leu |
|---|---|---|---|
| N 1/4 | 0.0 | 2.698354914233508 | 189.35537563619408 |
| N 1/2 | 0.0 | 15.961241098025225 | 252.52052674091883 |
| N 3/4 | 0.0 | 20.705119756968106 | 304.3360067260477 |
| EN | 0.0 | 19.077707649261214 | 334.21317474446505 |Figure S2
### Chart: ValCons (µM)
| Category |
|---|5.4%
6.4%
25.3%
32.2%
5.9%
269
38.0%
267
1.4%
212
30.6%
334
304
203
243
253
153
189
54
123
19
3
21
16
96
94
54
82
Valine*
Leucine
valine
leucine
MP
5.5
7.5
7.9
7.1
76
44.3
36.0
30.5
valine
leucine
24
55.7
64
69.5
6.0
10.1
9.9
8.8
pyruvate
α-acetolactate
α-ketoisovalerate
α-ketoisocaproate
0.5
2.6
2.0
1.7
0.0
0.5
0.3
0.3
N 1/4
N 1/2
N 3/4
EN
24
19.0
21.5
### Chart: Isobutanol (µM)
| Category | labelled | non labelled |
|---|---|---|
| N1/4 | 13.350438344391836 | 27.54537523928663 |
| N1/2 | 40.29703588800051 | 59.596459124395395 |
| N3/4 | 57.462764345040156 | 122.89745017172116 |
| EN | 98.09462563844197 | 520.408688601419 |36.5
### Chart: Isoamyl alcohol (µM)
| Category | labelled | non labelled |
|---|---|---|
| N1/4 | 0.24544277914246157 | 21.379471682748427 |
| N1/2 | 2.4993864941882387 | 44.13095854663711 |
| N3/4 | 2.958825990266825 | 54.88723442550688 |
| EN | 3.998729648803866 | 129.962062605932 |3.0%
5.1%
5.4%
1.1%
3
2
0
4
### Chart: Isobutyric acid (µM)
| Category | labelled | non labelled |
|---|---|---|
| N1/4 | 0.0 | 2.2263265269902286 |
| N1/2 | 1.1194804849150362 | 1.6112927433202027 |
| N3/4 | 0.7763044695994807 | 2.1974724681499187 |
| EN | 0.8802314099796399 | 4.39061535636551 |16.7%
26.1%
41.0%
0.0%
1
1
0
1
15.9%
520
31.9%
40.3%
60
123
32.6%
28
98
57
13
40

## Slide 3
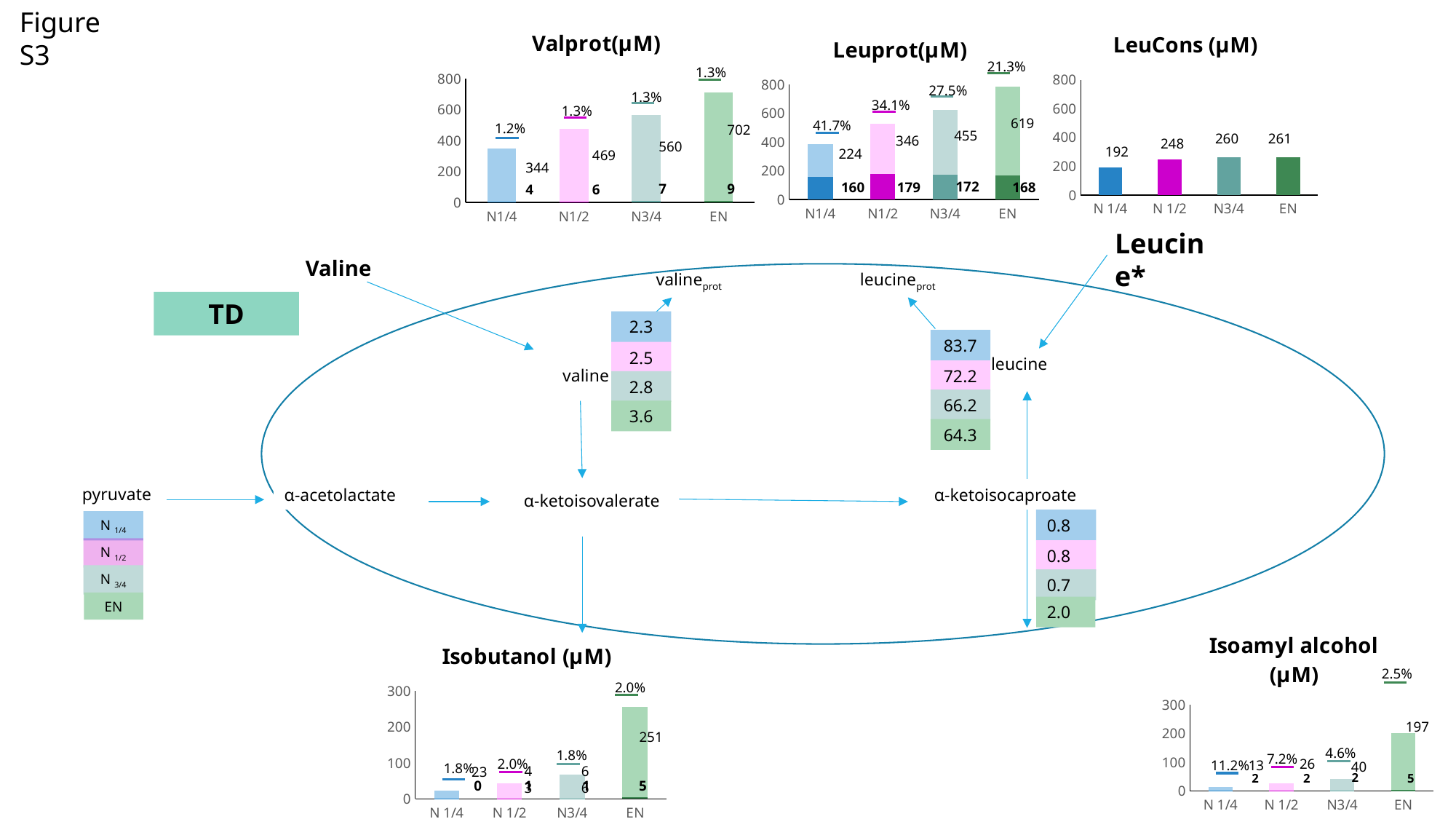

Figure S3
### Chart: Valprot(µM)
| Category | Labeled | Unlabeled |
|---|---|---|
| N1/4 | 4.315865736407822 | 343.7378226835778 |
| N1/2 | 6.108148881161525 | 469.2341765010583 |
| N3/4 | 7.319517368697513 | 560.0849298171562 |
| EN | 9.285860433912779 | 702.2743260728134 |1.3%
1.3%
1.3%
1.2%
702
560
469
344
7
9
4
6
### Chart: LeuCons (µM)
| Category | Consumed um |
|---|---|
| N 1/4 | 191.64038388645798 |
| N 1/2 | 248.19291980276827 |
| N3/4 | 260.437073728446 |
| EN | 260.9770992366412 |
### Chart: Leuprot(µM)
| Category | Labeled | Unlabeled |
|---|---|---|
| N1/4 | 160.39220836499123 | 224.47211884554562 |
| N1/2 | 179.2943355743869 | 346.4184208378308 |
| N3/4 | 172.38582961035797 | 454.69976373397816 |
| EN | 167.7255967079712 | 619.1643714270098 |21.3%
27.5%
34.1%
619
41.7%
455
260
261
346
248
192
224
172
179
168
160
Leucine*
Valine
valineprot
leucineprot
TD
2.3
83.7
2.5
leucine
valine
72.2
2.8
66.2
3.6
64.3
pyruvate
α-acetolactate
α-ketoisocaproate
α-ketoisovalerate
0.8
N 1/4
N 1/2
N 3/4
EN
0.8
0.7
2.0
### Chart: Isoamyl alcohol (µM)
| Category | labelled | non labelled |
|---|---|---|
| N 1/4 | 1.6238252599789686 | 12.881089969846567 |
| N 1/2 | 2.0138405440684584 | 25.995068274686183 |
| N3/4 | 1.94895004288343 | 40.009070256006346 |
| EN | 5.1319927696965575 | 197.31348138006905 |2.5%
197
4.6%
7.2%
26
11.2%
13
40
2
5
2
2
### Chart: Isobutanol (µM)
| Category | labelled | non labelled |
|---|---|---|
| N 1/4 | 0.4397368727821235 | 23.459006213202848 |
| N 1/2 | 0.8902942510491771 | 43.07485394891019 |
| N3/4 | 1.2242087170367426 | 65.85572098360667 |
| EN | 5.13409271584264 | 250.93038188977536 |2.0%
1.8%
2.0%
1.8%
0
1
1
5
251
43
66
23

## Slide 4
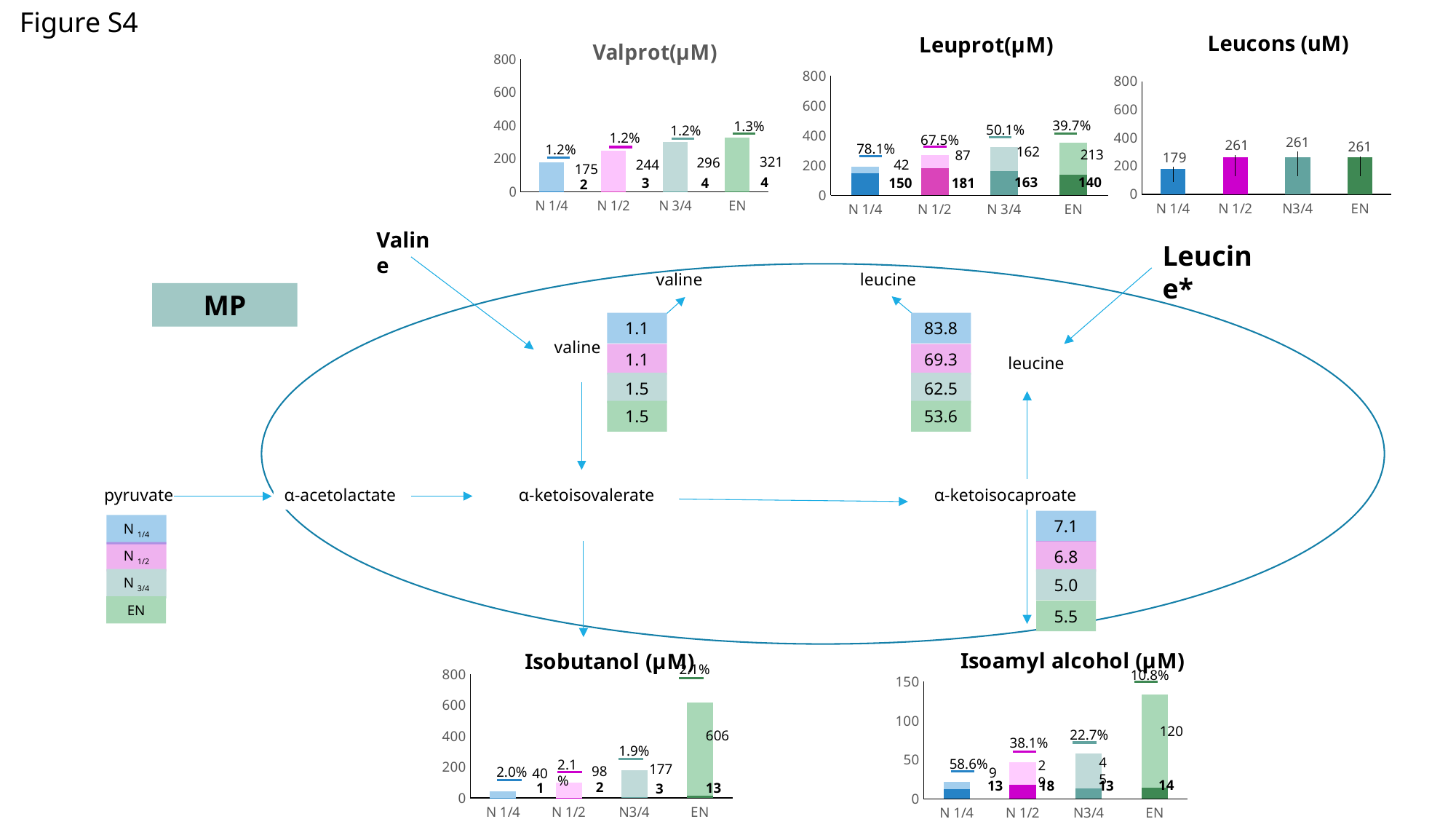

Figure S4
### Chart: Leuprot(µM)
| Category | labelled Leu | proteinogenic Leu |
|---|---|---|
| N 1/4 | 149.9747581868289 | 42.078972363598695 |
| N 1/2 | 181.1580728493275 | 87.32369498961654 |
| N 3/4 | 162.7155879173977 | 162.32553856561807 |
| EN | 140.32713848678807 | 212.96374390693816 |
### Chart: Leucons (uM)
| Category | consumed uM |
|---|---|
| N 1/4 | 178.83126006310903 |
| N 1/2 | 260.9770992366412 |
| N3/4 | 260.9770992366412 |
| EN | 260.9770992366412 |
### Chart: Valprot(µM)
| Category | labelled val | proteinogenic |
|---|---|---|
| N 1/4 | 2.052632562428041 | 174.89845040550657 |
| N 1/2 | 2.955771320666879 | 244.38910906986695 |
| N 3/4 | 3.698120372570585 | 295.7448247748452 |
| EN | 4.1337625847857025 | 321.35935432747436 |39.7%
1.3%
50.1%
1.2%
1.2%
67.5%
78.1%
1.2%
162
213
87
321
296
42
244
175
163
140
4
150
181
4
3
2
Valine
Leucine*
valine
leucine
MP
1.1
1.1
1.5
1.5
83.8
69.3
62.5
53.6
valine
leucine
pyruvate
α-acetolactate
α-ketoisovalerate
α-ketoisocaproate
7.1
6.8
5.0
5.5
N 1/4
N 1/2
N 3/4
EN
### Chart: Isobutanol (µM)
| Category | labelled | non labelled |
|---|---|---|
| N 1/4 | 0.8158714809943854 | 40.07994210268408 |
| N 1/2 | 2.0628006720059755 | 97.83069434038993 |
| N3/4 | 3.3456819792859225 | 177.0145325374754 |
| EN | 12.803018604765121 | 605.7002956350958 |2.1%
606
1.9%
2.1%
177
98
2.0%
40
2
13
1
3
### Chart: Isoamyl alcohol (µM)
| Category | labelled | non labelled |
|---|---|---|
| N 1/4 | 12.667874891775682 | 8.957039570115207 |
| N 1/2 | 17.75217235704221 | 28.878172683783138 |
| N3/4 | 13.102132684172743 | 44.74392773160096 |
| EN | 14.434275365447792 | 119.52651688928809 |10.8%
120
22.7%
38.1%
45
58.6%
29
9
14
13
13
18
